# Role of molecular conformations in rubrene polycrystalline films growth from vacuum deposition at various substrate temperatures

**DOI:** 10.1038/srep40824

**Published:** 2017-01-16

**Authors:** Ku-Yen Lin, Yan-Jun Wang, Ko-Lun Chen, Ching-Yuan Ho, Chun-Chuen Yang, Ji-Lin Shen, Kuan-Cheng Chiu

**Affiliations:** 1 Department of Physics and Center for Nanotechnology, Chung Yuan Christian University, Chungli District, Taoyuan City, 32023, Taiwan; 2 Department of Mechanical Engineering, Chung Yuan Christian University, Chungli District, Taoyuan City, 32023, Taiwan

## Abstract

We report on the optical and structural characterization of rubrene polycrystalline films fabricated from vacuum deposition with various substrate temperatures (*T*_*sub*_). Depending on *T*_*sub*_, the role of twisted and planar rubrene conformational isomers on the properties of rubrene films is focused. The temperature (*T*)-dependent inverse optical transmission (IOT) and photoluminescence (PL) spectra were performed on these rubrene films. The origins of these IOT and PL peaks are explained in terms of the features from twisted and planar rubrene molecules and of the band characteristics from rubrene molecular solid films. Here, two rarely reported weak-peaks at 2.431 and 2.605 eV were observed from IOT spectra, which are associated with planar rubrene. Besides, the *T*-dependence of optical bandgap deduced from IOT spectra is discussed with respect to *T*_*sub*_. Together with IOT and PL spectra, for *T*_*sub*_ > 170 °C, the changes in surface morphology and unit cell volume were observed for the first time, and are attributed to the isomeric transformation from twisted to planar rubrenes during the deposition processes. Furthermore, a unified schematic diagram in terms of Frenkel exciton recombination is suggested to explain the origins of the dominant PL peaks performed on these rubrene films at 15 K.

Motivated by a high hole mobility^1^,^2^,^3^,^4^ and a long exciton diffusion length^5^,^6^ observed in rubrene (C_42_H_28_) single crystal, an intense research effort has been devoted to the preparation and characterization of various rubrene molecular solid films suitable for applications in organic electronic and optoelectronic devices^7^,^8^,^9^,^10^,^11^,^12^,^13^,^14^,^15^,^16^. Rubrene molecule consists of a tetracene backbone with four phenyl side rings, and each side ring lies in a plane nearly perpendicular to the tetracene backbone. The rubrene molecule has two conformations: one with a twisting tetracene-backbone of about 42° (hereafter referred to as a twisted rubrene) and another one with a planar tetracene backbone (referred to as a planar rubrene). For a free rubrene molecule, the total energy of a twisted rubrene is smaller than that of a planar rubrene by about 163~210 meV based on the electronic structures calculation^8^,^17^,^18^. Thus, in vapor phase or in thin amorphous films, rubrene molecules possess the low energy state of twisting conformation; however in highly crystalline films or single crystals, rubrene molecules adopt a planar conformation in which the energy required to planarize the backbone is compensated by the lattice energy^8^,^13^,^17^,^18^,^19^,^20^,^21^,^22^,^23^. The rubrene molecular solids are formed by weak intermolecular interactions, and hence different growth methods can lead to different polymorphs^24^. The rubrene crystalline grains growth by vapor condensation exhibit a face-centered orthorhombic structure^8^,^9^,^11^,^15^,^24^. The high mobility observed in rubrene single crystal^1^,^2^ is attributed to the slipped-cofacial configuration along the long molecular-axis between two adjacent planar rubrene molecules, and this cofacial π-stack interactions significantly enhances the intermolecular charge transfer integral^3^.

For organic molecular solids formed by weak intermolecular interactions, the properties of the constituent molecules are only slightly changed with respect to those of a free molecule (which are determined by the strong intramolecular interactions). Thus, some of the features observed in a molecular solid can be traced back to the features observed in a free molecule^25^. The energy levels of a free rubrene molecule^18^,^19^,^20^,^22^ and the electronic band structure of the rubrene crystal^3^,^26^ have been theoretically calculated and experimentally confirmed by optical characterization methods for the cases of rubrene molecules dissolved in various solvents^18^,^20^,^27^,^28^,^29^,^30^,^31^,^32^, rubrene thin films^18^,^23^,^30^,^32^,^33^, and rubrene single crystals^27^,^28^,^29^,^30^,^31^,^33^,^34^. However, relatively little attention has been paid to the discussion on the role of conformational change in the rubrene molecular solid films fabricated from different growth conditions.

Though rubrene single crystal possesses excellent characters^1^,^2^,^3^,^4^,^5^,^6^,^7^,^8^,^9^, the high-cost and difficulty of fabricating large-area and highly crystalline rubrene thin films limit its application in practical devices. Thus, an intense research effort has been devoted to find suitable methods of depositing rubrene polycrystalline films with similar characters^10^,^15^,^16^,^17^,^18^. Recently, by using a vacuum deposition method, we reported a series of rubrene polycrystalline films fabricated under a fixed source temperature (*T*_*sou*_) of 300 °C and with substrate temperature (*T*_*sub*_) varied from 103 to 221 °C^12^. The growth behaviors and structural properties for these as-deposited rubrene films exhibit a strong *T*_*sub*_-dependence, which suggests that more attention should be paid to the correlations between the fabrication process and the properties of the as-prepared rubrene solid films for use in organic electronic and optoelectronic devices. In this paper we present the optical and structural characterization of the rubrene polycrystalline films fabricated from vacuum deposition with various *T*_*sub*_ and focus on the role of twisted and planar rubrene conformational isomers on the properties of these rubrene films.

## Results and Discussion

**Figure 1:**
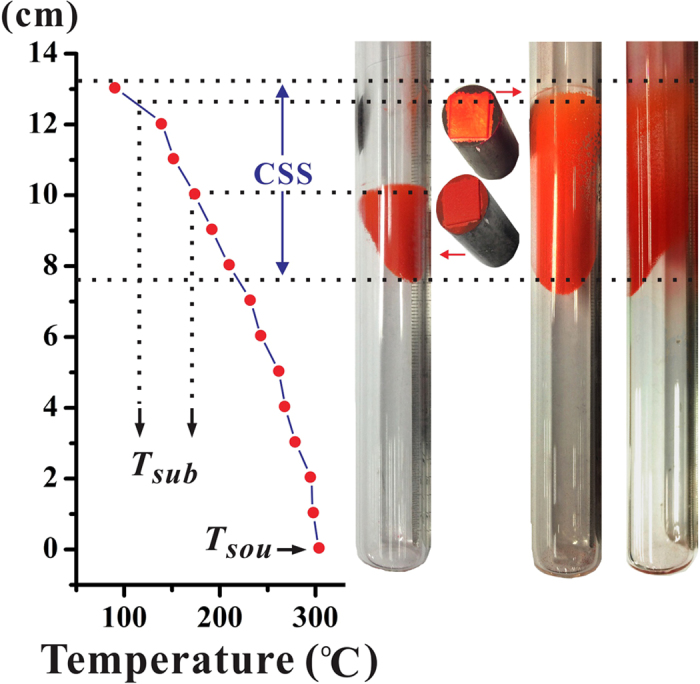
Growth ampoule with CSS region of rubrene corresponds to *T*_*sou*_ = 300 °C. Two rubrene polycrystalline films (on indium-tin-oxide substrate with graphite susceptor) deposited with different *T*_*sub*_ are illustrated.

Figure 1 demonstrates the constitutional super-saturation (CSS) phenomenon^12^,^35^ observed on the sidewall of the growth ampoule along the mass transport direction with *T*_*sou*_ = 300 °C. The CSS region is a place where the actual vapor pressure (from the source side) exceeds the equilibrium vapor pressure sufficiently to allow for nucleation and subsequent growth, and hence the growth in this region is dominated by surface kinetics controlled regime. The CSS region can be utilized either for depositing films with high-quality crystalline grains or even for growing single crystals. According to the distribution of CSS region as depicted in Fig. 1, *T*_*sub*_ was varied from 103 to 221 °C. Then, an indium-tin-oxide (ITO) coated glass was chosen as the substrate and adhered underneath a cylindrical graphite susceptor. By adjusting the substrate position, *T*_*sub*_ can be controlled. Within the CSS region, the choice of *T*_*sub*_ not only plays an important role in the growth rate but also has a strong impact on the structural properties of the as-deposited rubrene films^12^.

### Surface morphology of rubrene films fabricated at various *T*_*sub*_

**Figure 2:**
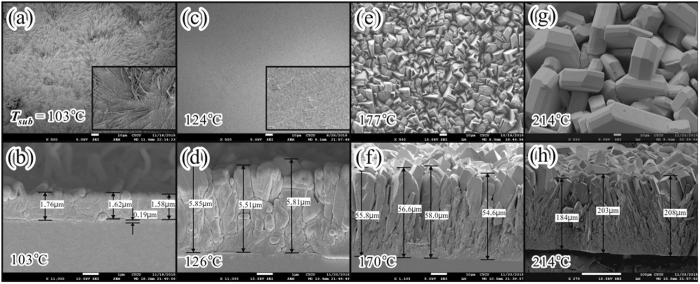
Top-view and side-view SEM images on the rubrene polycrystalline films deposited with various *T*_*sub*_.

From scanning electron microscopic (SEM) images as shown in Fig. 2, the film thickness as well as the average grain size of the as-deposited rubrene polycrystalline films increased with increasing *T*_*sub*_ due to the enhancement of adhesion coefficient and surface diffusion energy of the adsorbed rubrene molecules. For the lowest *T*_*sub*_ of 103 °C adopted in this work, the surface morphology exhibited kinetic roughness and some dendritic fibers with average diameter of 500 ± 250 nm were identified as presented in Fig. 2(a,b). As *T*_*sub*_ slightly increased to 124 °C, the density of the fibers reduced considerably as illustrated in Fig. 2(c,d). Furthermore, a distinct change in morphology was observed for *T*_*sub*_ ≧ 170 °C, in which the crystalline grains displayed remarkable facets as shown in Fig. 2(e–h). The morphologies of these highly crystalline rubrene grains growth from CSS region with high enough *T*_*sub*_ were similar to those reported results for single crystals growth from hot-wall deposition near thermal equilibrium conditions^8^,^9^,^10^,^11^.

### Structural characteristics of rubrene films fabricated at various *T*_*sub*_

**Figure 3:**
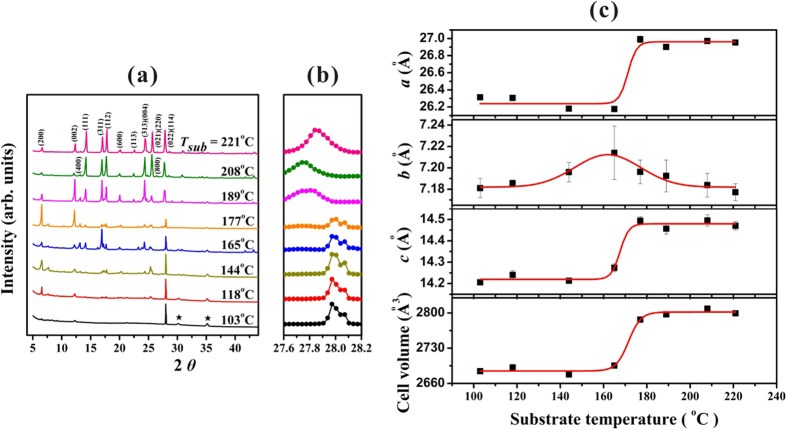
(**a**) XRD patterns on the rubrene polycrystalline films deposited with various *T*_*sub*_. The two peaks indicated by ★ are from ITO. (**b**) An enlarged view of the (022) peak at around 28° with an increment step of 0.03°. (**c**) The lattice constants of *a, b, c* and unit cell volume *V* fitted from GSAS.

To further investigate the *T*_*sub*_-dependence of the structural properties of the as-fabricated rubrene films, X-ray diffraction (XRD) analysis was applied. As demonstrated in Fig. 3(a), the rubrene crystalline grains growth from vacuum deposition exhibited a face-centered orthorhombic structure. By using the general structure analysis system (GSAS)^36^ and taking the standard *Cmca* setting^9^,^24^,^27^, the Miller indices of the diffraction peaks were assigned. In addition, from an enlarged view of the peak at about 28° as shown in Fig. 3(b) (note the space between two dots represents a scanning increment of 0.03°), a transformation was perceived for the rubrene films with *T*_*sub*_ ~ 170 °C. The fitted lattice constants of *a, b, c* and volume of unit cell *V* refined from GSAS were depicted in Fig. 3(c) with respect to *T*_*sub*_. It is interesting to note that an increase in *V* by 4% due to the increase of *a* and *c* was observed for *T*_*sub*_ > 170 °C. From Fig. 3(b), with increasing *T*_*sub*_ the reduction of the assigned (022) peak position indicates that the increase of *c* was estimated around 2%, which equally confirmed the change as illustrated in Fig. 3(c). Based on the measured values of *V* and taking a face-centered orthorhombic structure, the averaged mass density changed from 1.317 ± 0.005 g/cm^3^ for *T*_*sub*_ < 170 °C to 1.262 ± 0.003 g/cm^3^ for *T*_*sub*_ > 170 °C. The mass density of 1.317 g/cm^3^ matched reasonably well with the published data of 1.30 g/cm^3^ for the crystalline films composed of twisted rubrenes^8^, which suggests that the films fabricated with *T*_*sub*_ < 170 °C were composed of twisted rubrenes. The mass density of 1.262 g/cm^3^ for *T*_*sub*_ > 170 °C matched with another reported value of 1.269 g/cm^3^ for single crystals^24^, which is assumed to be composed of planar rubrenes.

### Inverse optical transmission spectroscopy

**Figure 4:**
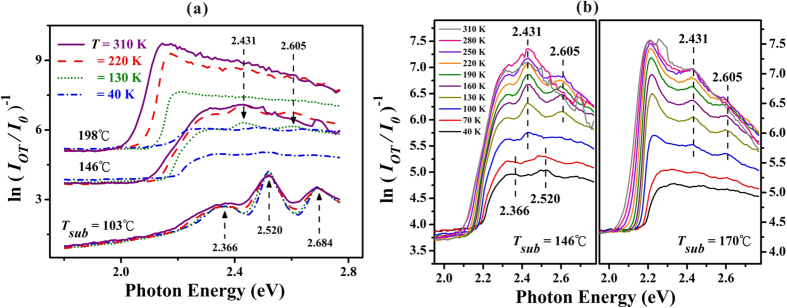
(**a**) IOT spectra performed at *T* = 40, 130, 220, and 310 K for rubrene films deposited with *T*_*sub*_ = 103, 146, and 198 °C. (**b**) Detailed *T*-dependence of IOT spectra for rubrene films deposited with *T*_*sub*_ = 146 and 170 °C.

To study the role of molecular conformations in the optical properties of rubrene films fabricated with various *T*_*sub*_, both optical transmission (OT) and photoluminescence (PL) were applied. For OT measurements through the rubrene films, if the reflection can be neglected, then the following approximate equations:


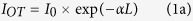



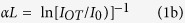


were adopted, where *α* is the absorption coefficient and *L* is the thickness of the films. Then from the plot of the inverse optical transmittance (IOT, *i.e*., ln[*I*_*OT*_/*I*_*0*_)]^−1^) versus incident photon energy *hv*, one can obtain the information about *α(hv*). Figure 4(a) shows the IOT spectra measured at *T* = 40, 130, 220, and 310 K for three rubrene films fabricated with *T*_*sub*_ = 103, 146, and 198 °C, separately. In this work, due to the fixed *T*_*sou*_ and fixed growth period, *L* of the as-deposited rubrene film increased with increasing *T*_*sub*_. Thus, at the same measurement temperature *T, I*_*OT*_ for thick rubrene films fabricated with high *T*_*sub*_ decreased and hence the corresponding IOT spectra shifted upward as depicted in Fig. 4(a).

### Absorption peaks for twisted versus planar rubrene molecules

The IOT spectra as shown in Fig. 4(a) could be categorized into three groups according to *T*_*sub*_. First, for the thin rubrene film fabricated at *T*_*sub*_ = 103 °C (with *L* ≈ 1.6 μm as depicted in Fig. 2), the IOT spectra exhibited three clear absorption peaks with energy positions of 2.366 ± 0.007, 2.520 ± 0.008, and 2.684 ± 0.009 eV. These error bars were estimated from the resolution of the monochromator used for IOT measurement. The energy positions for these three peaks were nearly independent of *T* except with a noticeable *T*-induced broadening for each peak. As listed in Table 1, these IOT peak positions obtained by this work matched well with those absorption peaks obtained for rubrene dissolved in various solvents^18^,^20^,^27^,^28^,^29^,^30^,^31^,^32^ and also for rubrene thin films^18^,^23^,^30^,^32^,^33^. The peak at 2.366 eV is assigned to the transition between the ground singlet state (S_0_) and the first excited singlet state (S_1_) in a twisted rubrene molecule, and the following two peaks of 2.520 and 2.684 eV are assigned to the vibrational progressions with vibrational energies of around 154 ± 15 and 164 ± 17 meV for the S_1_ state^18^,^20^,^27^,^28^,^29^,^30^,^31^,^32^. From the comparison of these works, we conclude that the constituent rubrene molecules within the thin rubrene films fabricated with lower *T*_*sub*_ still retain the twisted conformation as in the vapor phase. Second, for the thicker rubrene film fabricated with *T*_*sub*_ = 146 °C (with *L* ≈ 17 μm)^12^, two rarely reported weak peaks at 2.431 ± 0.008 and 2.605 ± 0.009 eV were identified at higher *T*, which were comparable to the results obtained by differential reflectance spectroscopy in rubrene ultra-thin molecular films on epitaxial graphene^23^. These two peaks are assigned to the S_0_ → S_1_ transition with different vibrational progressions for a planar rubrene molecule^23^, separately. Above experimental data suggest that the first vibrational energy of S_1_ state for planar rubrene (around 174 ± 17 meV) was slightly larger than that for twisted rubrene (154 ± 15 meV). Third, for the thick rubrene film fabricated with *T*_*sub*_ = 198 °C (with *L* ≈ 128 μm)^12^, the transmitting signals were too weak and only the background noise levels from the dark box were detected (which were close to the detecting limit of the optometer used). In such a case, the features from a free rubrene molecule vanished, but the characteristics from electronic band structure of the rubrene crystal occurred. The optical bandgap can be clearly determined from the absorption threshold, which will be discussed later. Besides, as depicted in Fig. 4(a) for the normalized IOT spectra of the thick film fabricated with *T*_*sub*_ = 198 °C performed at *T* = 220 and 310 K, the slow decrease after reaching a maximum was due to the normalization for the inverse of *I*_*OT*_/*I*_*0*_, in which the *I*_*OT*_ spectra approached to low-background levels but the *I*_*0*_ source spectrum from tungsten-halogen lamp decreased with increasing photon energy in this region. Furthermore, for those thick film fabricated with *T*_*sub*_ = 146 or 198 °C as shown in Fig. 4(a), beyond the absorption threshold the increase of IOT (*i.e*., the decrease of the transmittance) spectra with increasing *T* could be explained by the fact that the incident light is absorbed and scattered more effective due to an increase of the thermally excited vibrational modes in the constituent rubrene molecules. In contrast, for the thin film fabricated with *T*_*sub*_ = 103 °C, the IOT spectrum only exhibited a thermal broadening around each peak with increasing *T*.

To confirm the influence from the twisted and planar conformations on the optical properties of the as-fabricated rubrene molecular films, Fig. 4(b) depicts the detailed IOT spectra performed at *T* varied from 40 to 310 K for the second and third groups of rubrene films deposited with *T*_*sub*_ = 146 and 170 °C, respectively. For the second group of rubrene thicker film deposited with *T*_*sub*_ = 146 °C, the peak at 2.366 eV (which was a weak and broad peak as shown in Fig. 4(a) for a thin film deposited with *T*_*sub*_ = 103 °C) disappeared and the peak at 2.520 eV (a dominant peak in Fig. 4(a) with *T*_*sub*_ = 103 °C) barely detected due to the weak transmitting signals. However two additional weak peaks at 2.431 and 2.605 eV which are assigned to a planar conformation^23^ were observed at *T* > 100 K. Thus, these two conformational isomers co-existed in the second group of rubrene films. For the third group of rubrene thick film deposited with *T*_*sub*_ = 170 °C, the peaks assigned to a twisted rubrene (2.366 and 2.520 eV) almost vanished, but the two weak peaks corresponding to a planar rubrene^23^ were again observed at *T* > 100 K. Because the calculated energy difference of twisted and planar conformations is about 163~210 meV^8^,^17^,^18^, the thermal energy corresponding to *T*_*sub*_ during the deposition process is too small for the required transformation energy. Thus, the energy needed to planarize the twisted backbone is proposed to be compensated by the lattice energy from a more efficient packing of the planar rubrene molecules in the bulk^8^,^17^,^18^, which can be induced either from the critical thickness of the film^13^ or from the interaction with substrate^23^. Our experimental findings reveal that the third group of thick rubrene films fabricated with *T*_*sub*_ > 170 °C with crystalline grain size > 5 μm (as estimated from Fig. 2(e,f)) provided adequate surroundings for the formation of crystalline grains composed of planar rubrenes. Thus, in contrast to twisted rubrenes in the same orthorhombic structure, with *T*_*sub*_ > 170 °C the steric interactions initiated by the four phenyl side rings to preserve the slipped-cofacial configuration^3^ between two adjacent planar rubrenes could be the origins for the remarkable facets as displayed in Fig. 2(e–h) and also for the increment in *V* by 4% as observed in Fig. 3.

### *T*-dependence of optical bandgap for rubrene molecular films

**Figure 5:**
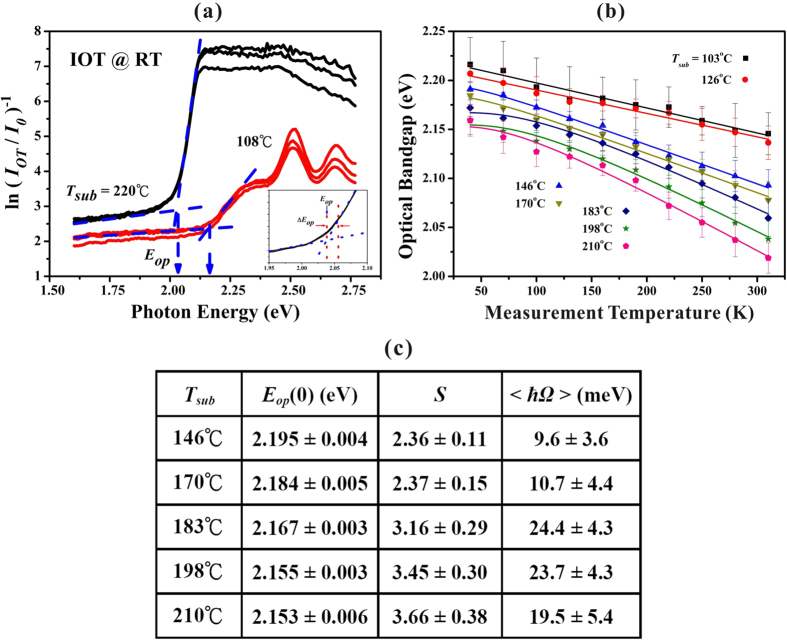
(**a**) Optical bandgap *E*_*op*_ determined from the IOT spectra for thin rubrene film (deposited at *T*_*sub*_ = 108 °C) and for thick rubrene film (*T*_*sub*_ = 220 °C), respectively. The inset shows the practical definition of Δ*E*_*op*_. (**b**) Variation of *E*_*op*_(*T*) for rubrene films deposited with various *T*_*sub*_ from 103 to 210 °C. The solid curves are the fitting results. (**c**) Fitting parameters of O’Donnell and Chen eq. for rubrene films fabricated with various *T*_*sub*_ = 146–210 °C.

As free molecules are brought together to make a molecular solid, the close packed of huge number of splitting electronic levels form a continuous electronic band^25^. In contrast to the strong covalent-bonded inorganic solids (*e.g*., Si and Ge), the weak intermolecular interactions in organic molecular solids only result in a small bandwidth of around 0.5~1.0 eV^25^, and the optical bandgap *E*_*op*_ is also expected to be sensitively dependent on the fabrication conditions (*e.g*., *T*_*sub*_). The magnitude of *E*_*op*_ can be experimentally deduced from the absorption threshold. Figure 5(a) demonstrates two IOT spectra performed at room temperature (RT) for thin and thick rubrene films fabricated with *T*_*sub*_ = 108 and 220 °C. The experiments on each sample were repeated three times and the data were well matched. As illustrated in Fig. 5(a), *E*_*op*_ determined from absorption threshold was practically defined at the intersection of two guide lines^37^, one for the transmitted background with *hv* ≪ *E*_*op*_ and the other one for the strong absorption with *hv* > *E*_*op*_. The error bar in *E*_*op*_ (*i.e*., Δ*E*_*op*_) was defined as shown in the inset of Fig. 5(a). The average values of *E*_*op*_(*T*) together with Δ*E*_*op*_(*T*) for rubrene films fabricated with various *T*_*sub*_ were depicted in Fig. 5(b). Various forms have been proposed to account for the physical significance of *E*_*op*_(*T*) which arises from the *T*-dependence of lattice dilatation and electron-phonon interaction. One of them proposed by O’Donnell and Chen^38^ as following,


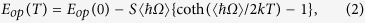


has been widely quoted, where *S* is a dimensionless electron-phonon coupling constant, <*ħΩ*> is an average energy of the interacting phonons, *kT* is the thermal energy, and the magnitude of {coth(<*ħΩ*>/2*kT*) − 1} represents the effective number of available phonons. The applications of Eq. (2) to some typical inorganic and organic semiconductors were discussed and summarized in our previous works^39^,^40^.

As depicted in Fig. 5(b), for the first group of thin rubrene films fabricated with *T*_*sub*_ = 103 and 126 °C, though *E*_*op*_ followed the trend to decrease with increasing *T*, due to the weak coupling the experimental range of *T* from 40 to 310 K was still not wide enough to give reasonable fitting parameters for the least-squares fit to Eq. (2). Next, for the second and third groups of rubrene films fabricated with *T*_*sub*_ ≧ 146 °C, the least-squares fit of these data gave reasonable parameters as listed in Fig. 5(c). Typically, in forming organic molecular solid films, the surface diffusion energy of the adsorbed molecules increases with increasing *T*_*sub*_, which in a certain sense can also enhance the compactness of the as-fabricated molecular films. Thus, both the coupling constant *S* and the average phonon energy <*ħΩ*> increased with increasing *T*_*sub*_. However, the compactness can also cause stronger intermolecular interactions to result in a slightly wider (conduction or valence) bandwidth and hence *E*_*op*_(0) decreased with increasing *T*_*sub*_ until a saturation is approached. As shown in Fig. 5(c), for the second group of rubrene films fabricated with *T*_*sub*_ = 146 and 170 °C, smaller values of *S* = 2.4 ± 0.1 and <*ħΩ*> = 10 ± 4 meV were obtained; whereas for the third group of thick rubrene films fabricated with *T*_*sub*_ = 183 to 210 °C, strong couplings with larger values of *S* = 3.4 ± 0.5 and <*ħΩ*> = 23 ± 6 meV were found. According to the results discussed above, the thick polycrystalline rubrene films fabricated with *T*_*sub*_ > 170 °C were composed of planar rubrenes dominantly. Thus, the analyzed results from Eq. (2) indicate that the grains formed by planar rubrenes possessed stronger electron-phonon coupling with higher phonon energy modes than those grains consisted of the mix of twisted and planar rubrene molecules.

### Photoluminescence analysis of rubrene films fabricated at various *T*_*sub*_

**Figure 6:**
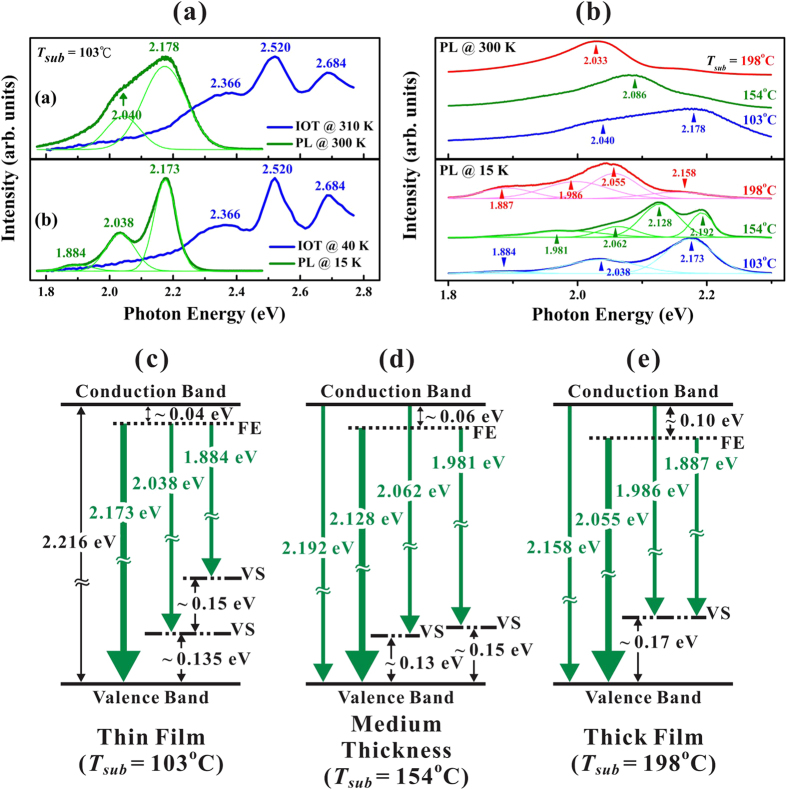
(**a**) Comparison of the normalized PL and IOT spectra performed at low and high *T* for the thin rubrene film deposited with *T*_*sub*_ = 103 °C. (**b**) Comparison of the PL spectra performed at 15 and 300 K on the rubrene films fabricated with *T*_*sub*_ = 103, 154 and 198 °C, respectively. (**c**) Schematic diagrams illustrating the origins of all the PL peaks performed at 15 K for the rubrene films fabricated with *T*_*sub*_ = 103 °C (which was composed of twisted rubrene), 154 °C (composed of both twisted and planar rubrene together with formation of band characteristics) and 198 °C (composed of planar rubrene with formation of band characteristics), respectively. FE: Frenkel exciton recombination and VS: vibrational state.

Complementary to OT with a bandwidth of 1.5 nm, a high resolution PL with a bandwidth of 0.3 nm was carried out. Figure 6(a) compares the normalized PL and IOT spectra performed at low and high *T* for the first group of thin rubrene film fabricated with *T*_*sub*_ = 103 °C, separately. The PL and IOT spectra performed at low *T* both depicted pronounced vibrational progressions that were dominated by three peaks, and their amplitude variation can be explained by the Franck-Condon principle^20^. As shown in Table 1, the three IOT peaks of 2.366, 2.520, and 2.684 eV matched well to those in the published results^18^,^20^,^27^,^28^,^29^,^30^,^31^,^32^ and were assigned to the twisted rubrene molecular characters; but the three PL peaks of 1.884 ± 0.003, 2.038 ± 0.002, and 2.173 ± 0.002 eV were about 0.07 eV lower than those published data for twisted rubrene in various solvents^20^,^27^,^31^,^32^. This difference is due to the solvent effects in solutions and the weak intermolecular interactions in these thin rubrene films. Besides, the big separation of the IOT and PL spectra as depicted in Fig. 6(a) and also from a comparison in Table 1 suggests that a rather large Stokes shift was observed for a twisted rubrene molecule, which was theoretically predicted from the unresolved vibrational progression involving low-frequency modes caused by the inductive effect of the phenyl substituents in the excited state^20^. This local molecular excited state commonly observed in organic molecular solids can be regarded as a Frenkel exciton (FE) which is the electron-hole pair localized on the same molecule^25^. Before recombination these excitons can diffuse and usually be trapped into a region of lower energy states nearby the defects, impurities, or grain boundary. Thus, PL emission from FE being trapped within a lower energy region can further generate a larger Stokes shift. However, the first and second vibrational energies of S_0_ state as deduced from our PL spectra at *T* = 15 K corresponding to twisted rubrene were estimated around 135 ± 4 and 154 ± 5 meV, which were similar to those published data from twisted rubrene in various solvents^20^,^27^,^31^,^32^ as listed in Table 1. With increasing *T* to effectively enhance the population of thermal vibrational modes, these peaks become broader as depicted in the spectrum performed at 300 K. Nevertheless, the first vibrational energy of 138 ± 4 meV deduced at 300 K still well matched to the one of 135 ± 4 meV at 15 K. This nearly *T*-independence of the vibrational energies (deduced from PL and absorption spectra for thin rubrene films and for rubrene in various solvents) is due to the strong intramolecular interactions of the constituent rubrene molecules.

Figure 6(b) compares the normalized PL spectra performed at 15 and 300 K on the three groups of rubrene polycrystalline films deposited with *T*_*sub*_ = 103, 154 and 198 °C, respectively. Under a similar pumping power, the PL intensity from the first group of thin rubrene film fabricated with *T*_*sub*_ = 103 °C was much larger than that from the third group of thick rubrene film with *T*_*sub*_ = 198 °C, which can be clearly distinguished with the naked eye. This finding reveals that the PL emission efficiency from twisted rubrenes was much larger than that from planar rubrenes. This is an important issue to be considered for their applications in organic light-emitting diodes. After a multiple Gaussian fit to each spectrum, the energy position for each peak in Fig. 6(b) was assigned. Comparing the spectra performed at 15 and 300 K, the thermal broadening effects observed at 300 K smeared the fine features observed at 15 K. Thus, hereafter we confine our discussion to the spectra performed at 15 K. For the PL on the first group of thin film fabricated with *T*_*sub*_ = 103 °C, three peaks of 1.884, 2.038 and 2.173 eV were correlated with twisted rubrenes as discussed early. From Fig. 5(b), for thin film fabricated with *T*_*sub*_ = 103 °C, *E*_*op*_ = 2.216 ± 0.028 eV at *T* = 40 K, thus the dominant PL peak of 2.173 eV was smaller than *E*_*op*_ by around 0.04 eV, and hence an emission via FE recombination was assumed as mentioned above. The other two peaks were assigned to the corresponding vibrational progressions with vibrational state (VS) energies of around 0.135 and 0.15 eV. A schematic diagram illustrating the origins of these three PL peaks observed for the first group of rubrene thin films was proposed as shown in the left-side on Fig. 6(c) with bold-green line representing the dominant PL peak and thin-green lines for the other two.

As shown in Fig. 6(b) for the third group of thick rubrene film fabricated with *T*_*sub*_ = 198 °C, four Gaussian peaks with energies of 1.887 ± 0.005, 1.986 ± 0.013, 2.055 ± 0.003 and 2.158 ± 0.003 eV were fitted. Indeed, as compared to *E*_*op*_(0) of 2.155 ± 0.003 eV as shown in Fig. 5(c), the PL emission at 2.158 eV could be assigned to the band-to-band transition. For the thick rubrene films composed of crystalline grains fabricated with high *T*_*sub*_ (see Fig. 2(e–h)), the characteristics from electronic band structure should leave their imprints on the optical spectra (*e.g*., see Fig. 4(a) for a strong absorption corresponding to an optical bandgap). Thus, the band-to-band emission is possible, though this PL peak at 2.158 eV is a weak shoulder barely distinguished from the spectra as shown in Fig. 6(b). However, the dominant PL peak at 2.055 eV (by a bold-green line) was still assigned through a FE recombination with energy lower than the corresponding *E*_*op*_ (2.155 eV) by around 0.10 eV. The other two PL peaks of 1.887 and 1.986 eV were separately assigned to the vibrational progressions of the above-mentioned peaks of 2.055 and 2.158 eV with VS energy of around 0.17 eV, as depicted in the right-side on Fig. 6(c). Because the third group of thick films was composed of planar rubrenes in contrast to the first group of thin films which was composed of twisted rubrenes, so these two conformational isomers could possess different FE recombination energies as well as different VS energies. For the second group of rubrene film deposited with *T*_*sub*_ = 154 °C, in addition to the band-to-band transition peak at *E*_*op*_ = 2.192 ± 0.002 eV, the dominant PL peak of 2.128 ± 0.002 eV (by bold-green line) was again governed via a FE recombination with energy lower than the corresponding *E*_*op*_ by around 0.06 eV. Similarly, the other two PL peaks of 2.062 ± 0.003 and 1.981 ± 0.002 eV could be assigned to the vibrational progressions from the peaks of 2.192 and 2.128 eV with various VS energies of around 0.13 and 0.15 eV as depicted in the middle on Fig. 6(c), respectively. Because of the co-existence of two isomers, two VS energies were introduced.

## Conclusion

Optical and structural properties of rubrene polycrystalline films growth from vacuum deposition under a fixed *T*_*sou*_ of 300 °C and with various *T*_*sub*_ from 103 to 221 °C have been studied. Depending on *T*_*sub*_, the as-fabricated rubrene polycrystalline films comprise two (twisted and planar) conformational rubrene isomers and the role of molecular conformations in the optical and structural properties of rubrene films is focused. Basically, these rubrene films can be categorized into three groups. For the first group of thin rubrene films fabricated with low *T*_*sub*_ ( = 103~126 °C), the constituent rubrene molecules within the small grains in these films retain the twisted conformation as in the vapor phase. From IOT spectra, three absorption peaks with energy positions of 2.366, 2.520, and 2.684 eV were observed. The peak at 2.366 eV is assigned to the S_0_ → S_1_ transition in a twisted rubrene, and the following two peaks of 2.520 and 2.684 eV are assigned to its vibrational progressions. The band characteristics of these thin organic films are not robust enough and the IOT and PL spectra are dominant by the twisted rubrene molecular features. For the second group of rubrene films fabricated with intermediate *T*_*sub*_ ( = 146~154 °C), the co-existence of two types of isomers were observed from the *T*-dependent IOT spectra in the rubrene films of medium thickness. Two additional weak peaks of 2.431 and 2.605 eV observed in the IOT spectra are assigned respectively to the S_0_ → S_1_ transition and to its vibrational progression for a planar rubrene molecule. For the second and third groups of rubrene films fabricated with *T*_*sub*_ = 146~221 °C, the electronic band characteristics of these molecular solid films formed by weak intermolecular interactions were clearly observed and the variation of *E*_*op*_(*T*) deduced from IOT spectra is determined and discussed with respect to *T*_*sub*_. Furthermore, from the PL measurement performed at 15 K, the dominant peak in each spectrum is suggested to be governed by the FE recombination, and a schematic diagram is planned to explain the origins of all the PL peaks observed in these three groups of rubrene films. Besides, for the third group of thick films fabricated with *T*_*sub*_ > 170 °C, both the remarkable facets in highly crystalline grains observed from SEM images and the increment in *V* by 4% evaluated from XRD data are proposed to be related to the abovementioned isomeric transformation from twisted to planar rubrenes. Above experimental findings suggest that the physical properties of rubrene molecular solid films are sensitively dependent on the conformation of the constituent molecules. Thus, more attention should be paid to the isomeric transformation during the deposition of rubrene molecular solid films for their future applications in organic electronic and optoelectronic devices.

## Methods

### Fabricating of rubrene polycrystalline films

As shown in Fig. 1, the growth ampoule was made of quartz tube with an inner diameter of 16 mm, an outer diameter of 20 mm, and a length of 30 cm. An ITO coated glass was chosen as the substrate and adhered underneath a cylindrical graphite susceptor with an outer diameter of 15 mm. Rubrene source powder (sublimed grade with purity >99.0% from Uni-Onward Corp., Taiwan) was used as received and placed at the bottom of the ampoule. By adjusting the positions of the source site and the substrate site relative to the temperature profile of a vertical furnace, *T*_*sou*_ and *T*_*sub*_ can be controlled separately. After a typical cleaning process, the whole growth ampoule was assembled together with 30~50 mg rubrene source powder. The growth ampoule was first under dynamic vacuum for 1.0 h at room temperature and then placed into the vertical furnace. The deposition period was kept for 1.0 h under dynamic vacuum condition. A further detailed description of this vacuum deposition process has been provided elsewhere^12^.

### Characterization

OT through the rubrene films was performed by using a scanning monochromator (ARC SpecrroPro-500) with a tungsten-halogen lamp as the light source. After a long-pass filter of 400 nm to eliminate the higher-order components, the monochromatic light with wavelength varied from 700 to 450 nm was focused on the sample. The increment of each step was set to 2 nm and the corresponding resolution was ±1.5 nm. The optical transmittance intensity *I*_*OT*_ as well as the original incident light intensity *I*_*0*_ was measured by an optometer (Graseby UDT S370) with wavelength correction. Then, the normalized OT spectrum was accomplished by dividing *I*_*OT*_ by *I*_*0*_ at each photon energy position. PL spectrum was performed with exciting by a focused diode laser of 405 nm on the rubrene films. The collected luminescence was dispersed by a spectrometer (Jobin Yvon/Spex TRIAX 550) and detected with a photomultiplier tube. The wavelength was scanned from 700 to 520 nm with an increment of 0.4 nm and a resolution of ±0.3 nm. The temperature of the sample site during the OT and PL measurements was controlled by a cryostat system. The morphology and crystallinity of the as-deposited rubrene polycrystalline films were examined by using a JOEL JSM-6335F scanning electron microscopy and by X-ray diffraction analysis with a Panalytical X’Pert Pro PW 3040/60 diffractometer, respectively.

## Additional Information

**How to cite this article:** Lin, K.-Y. *et al*. Role of molecular conformations in rubrene polycrystalline films growth from vacuum deposition at various substrate temperatures. *Sci. Rep.* **7**, 40824; doi: 10.1038/srep40824 (2017).

**Publisher's note:** Springer Nature remains neutral with regard to jurisdictional claims in published maps and institutional affiliations.

## Figures and Tables

**Table 1:** Comparison of the peaks observed from PL and absorption spectra for twisted rubrene molecules in various solvents and for thin films composed of twisted rubrene molecules.

Rubrene in various solvents								
Ref. cited in this work	PL peaks (eV)			Absorption peaks (eV)				Note
18				2.37	2.53	2.62		in acetone
20	1.97	2.12	2.25	2.36	2.52	2.68		in cyclohexane
27	1.96	2.09	2.23	2.35	2.52	2.68	2.87	in chloroform
28				2.34	2.50	2.65	2.84	in chloroform
29		2.11	2.23					in acetone
30				2.35	2.52	2.68	2.85	in chloroform
30				2.37	2.53	2.69	2.85	in acetone
31	1.94	2.10	2.21	2.36	2.53	2.70	2.82	in toluene
32		2.13	2.25	2.35	2.51	2.68	2.85	in toluene
**Rubrene thin films**								
18				2.33	2.51	2.66		@ RT
23				2.33	2.49	2.66		by DRS *in situ*
30				2.34	2.51	2.67	2.85	amorphous film @ RT
32				2.34	2.50	2.67	2.85	amorphous film @ 77 K
33				2.34	2.51	2.69		@ RT
This work	1.88	2.04	2.17	2.37	2.52	2.68		PL @ 15 K; IOT @ 40 K
